# Representativeness of breast cancer cases in an integrated health care delivery system

**DOI:** 10.1186/s12885-015-1696-9

**Published:** 2015-10-14

**Authors:** Scarlett Lin Gomez, Salma Shariff-Marco, Julie Von Behren, Marilyn L. Kwan, Candyce H. Kroenke, Theresa H. M. Keegan, Peggy Reynolds, Lawrence H. Kushi

**Affiliations:** 1 Cancer Prevention Institute of California, 2201 Walnut Avenue, Suite 300, Fremont, CA 94538 USA; 2 Cancer Prevention Institute of California, 2001 Center Street, Suite 700, Berkeley, CA 94704 USA; 3 Department of Health Research and Policy, School of Medicine, Stanford, 94305 CA USA; 4 Division of Research, Kaiser Permanente Northern California, 2000 Broadway, Oakland, CA 94612 USA

**Keywords:** Cancer research network, Electronic medical records, Electronic health records, Comparative effectiveness research, NCI-designated cancer center, Breast cancer

## Abstract

**Background:**

Integrated health care delivery systems, with their comprehensive and integrated electronic medical records (EMR), are well-poised to conduct research that leverages the detailed clinical data within the EMRs. However, information regarding the representativeness of these clinical populations is limited, and thus the generalizability of research findings is uncertain.

**Methods:**

Using data from the population-based California Cancer Registry, we compared age-adjusted distributions of patient and neighborhood characteristics for three groups of breast cancer patients: 1) those diagnosed within Kaiser Permanente Northern California (KPNC), 2) non-KPNC patients from NCI-designated cancer centers, and 3) those from all other hospitals.

**Results:**

KPNC patients represented 32 % (*N* = 36,109); cancer center patients represented 7 % (*N* = 7805); and all other hospitals represented 61 % (*N* = 68,330) of the total breast cancer patients from this geographic area during 1996–2009. Compared with cases from all other hospitals, KPNC had slightly fewer non-Hispanic Whites (70.6 % versus 74.4 %) but more Blacks (8.1 % versus 5.0 %), slightly more patients in the 50–69 age range and fewer in the younger and older age groups, a slightly lower proportion of *in situ* but higher proportion of stage I disease (41.6 % versus 38.9 %), were slightly less likely to reside in the lowest (4.2 % versus 6.5 %) and highest (36.2 % versus 39.0 %) socioeconomic status neighborhoods, and more likely to live in suburban metropolitan areas and neighborhoods with more racial/ethnic minorities. Cancer center patients differed substantially from patients from KPNC and all other hospitals on all characteristics assessed. All differences were statistically significant (*p* < .001).

**Conclusions:**

Although much of clinical research discoveries are based in academic medical centers, patients from large, integrated medical centers are likely more representative of the underlying population, providing support for the generalizability of cancer research based on electronic data from these centers.

## Background

Integrated health care delivery systems, such as those within the National Cancer Institute (NCI)-funded Cancer Research Network [1, 2], have expansive and integrated electronic medical records (EMRs), and are well-poised to conduct research that leverages the detailed clinical and outcomes data within EMRs [3, 4]. The use of EMRs can facilitate generation of important insights in cancer control research, including cancer survivorship research [5, 6], health services and comparative and cost effectiveness research, cancer epidemiology, health promotion, and cancer communication and medical care decision-making, in an expedient and cost-effective manner [1, 2, 5, 6]. Because of the generally broad population coverage of these integrated health care delivery systems, they have the potential to produce findings that are generalizable to the population. However, current information regarding the representativeness of clinical populations from these integrated health care delivery systems is limited, and thus the generalizability of research findings to the overall population is uncertain, particularly in cancer control research.

To determine whether clinical populations from a large integrated health care delivery system are sociodemographically and clinically representative of the general population of breast cancer patients in California, we compared patient demographic and social and built environment neighborhood characteristics for breast cancer patients diagnosed within the Kaiser Permanente Northern California (KPNC) health care delivery system (a member of the CRN) with non-KPNC patients in the same underlying geographic region. Because much of clinical cancer research discoveries are based in academic medical centers, we also assessed representativeness of KPNC breast cancer patients relative to those at NCI-designated cancer centers in the Northern California region. We focused on breast cancer as it is the most commonly-diagnosed cancer among women from all major racial/ethnic groups in the Northern California population. In addition to patient demographic and clinical characteristics, we were particularly interested in comparing differences in social and built environment factors given recent initiatives to incorporate neighborhood and multilevel data into cancer research [7–10].

## Methods

We selected all female *in situ* and invasive breast cancer cases (ICD-O-3 C500–509) reported to the population-based California Cancer Registry (CCR), a part of the NCI’s Surveillance, Epidemiology, and End Results (SEER) Program. We included cases diagnosed from 1996 through 2009 and whose county of residence and reporting facility was within the KPNC catchment region, including the counties of Alameda, Amador, Contra Costa, El Dorado, Fresno, Madera, Marin, Napa, Placer, Sacramento, San Francisco, San Joaquin, San Mateo, Santa Clara, Solano, Sonoma, and Yolo. All cases were assigned to 2000 U.S. Census block groups based on residential addresses at the time of diagnosis. Patients (*n* = 7567 or 6 %) were excluded if their addresses did not match to a census tract/block group, have at least Zip + 4 address information, and/or were not assigned latitude/longitude coordinates. Among the cases excluded because of missing census tract information, the same percentage, 7 %, were from cancer centers as the tracted cases. The untracted cases were slightly less likely to be from KPNC than the cases with tract information (28 % versus 32 %). We did not obtain informed consent from the patients as we analyzed de-identified cancer registry data.

The reporting hospital for each patient is the hospital with the earliest admission date for that patient’s tumor, usually the diagnosing facility. These hospitals are categorized as a KPNC medical facility, a non-KPNC cancer center hospital, or a non-KPNC non-cancer center hospital. Cancer center hospitals were based on NCI cancer center designations as of April 2010 (http://www.cancer.gov/researchandfunding/extramural/cancercenters/find-a-cancer-center).

We linked patients’ block group of residence to census information from the 2000 Census Summary File 3 (SF-3). Block-group level neighborhood features included poverty level, an index of socioeconomic status (SES) based on seven Census indicators for education, occupation, unemployment, household income, poverty, rent, and house values [11]; Asian ethnic enclave; Hispanic ethnic enclave; racial/ethnic composition; population density; and urbanization [12, 13]. Ethnic enclaves are areas that maintain more cultural mores and are ethnically distinct from the surrounding area. Both indices of ethnic enclaves were developed using principal components analysis; the Hispanic ethnic enclave index includes Census data on linguistic isolation, English fluency, Spanish language use, Hispanic ethnicity, immigration history, and nativity [14, 15], and the Asian ethnic enclave index includes data on Asian/Pacific Islander race/ethnicity, language, nativity, and recency of immigration [16–19]. The SES and ethnic enclave indices were classified into quintiles based on their block group distributions in California. Urbanization is a composite measure based on census defined urbanized area, population size, and population density [12].

We compared the distributions (age-adjusted to the age distribution of all patients) of individual-level clinical, demographic, and neighborhood characteristics of the patients from KPNC reporting hospitals (referred to as “KPNC”) to those from non-KPNC cancer center reporting hospitals (referred to as “CC”), and non-KPNC non-cancer center reporting hospitals (referred to as “all other hospitals”). Testing for significant differences was conducted using the chi-squared test with Bonferroni family-wise error rate adjustment for 51 comparisons (3 groups × 17 variables), with an adjusted *p*-value threshold of *p* = .001. This project, involving analysis of de-identified data, was approved by the Institutional Review Board of the Cancer Prevention Institute of California, which waived the requirement for patient informed consent.

## Results

The final study sample consisted of 112,244 women diagnosed with breast cancer in the northern California study counties from 1996 through 2009 (Table 1). KPNC patients represented 32 % (*N* = 36,109), all other hospital patients represented 61 % (*N* = 68,330), and CC patients represented 7 % (*N* = 7805) of the total breast cancer patients during this time period. Compared with patients from all other hospitals, KPNC patients included a lower proportion of non-Hispanic Whites (70.6 % versus 74.4 %) but a higher proportion of non-Hispanic Blacks (8.1 % versus 5.0 %), had slightly more patients in the 50–69 age range and fewer in the younger and older age groups, had considerably more privately insured (92.4 % versus 52.7 %) and fewer publicly insured (2.5 % versus 24.8 %) patients, and had a slightly lower proportion of *in situ* (17.0 % versus 19.3 %) but a higher proportion of stage I (41.6 % versus 38.9 %) cases. KPNC patients had slightly higher proportions of lobular histology compared with patients from all other hospitals (17.2 % versus 14.3 %). During this time period, KPNC patients also had considerably lower proportions of unknown estrogen and progesterone receptor status than patients from all other hospitals (12.1 % unknown among KPNC cases versus 24.6 % unknown among patients from all other hospitals); thus the relative distributions of hormone receptor status could not be compared.

**Table 1:** Age-adjusted percent distribution of patient- and neighborhood-level characteristics by hospital type, females diagnosed with breast cancer, Northern California^a^, 1996–2009

Characteristic	KPNC (*N* = 36,109) %	Non-KPNC		All (*N* = 112,244) %
		All other hospitals (*N* = 68,330) %	Cancer centers (*N* = 7805) %	
Race				
Non-Hispanic white	70.6	74.4	71.1	73.0
Non-Hispanic black	8.1	5.0	6.5	6.0
Hispanic	7.5	7.0	5.4	7.0
Asian/Pacific Islander	13.0	12.6	16.0	13.0
Non-Hisp Am Indian/Alas Native	0.3	0.3	0.2	0.3
Other/unknown	0.6	0.7	0.7	0.6
Age at diagnosis				
< 30	0.3	0.4	0.8	0.4
30–39	3.6	4.6	7.2	4.5
40–49	16.9	18.9	23.1	18.5
50–59	26.1	24.3	27.5	25.1
60–69	25.3	20.6	20.4	22.1
70–79	19.0	19.1	13.7	18.7
80–89	7.8	10.6	6.5	9.4
90+	0.9	1.6	0.7	1.3
Insurance/payment source				
Any public/Medicaid/military	2.5	24.8	28.9	17.9
Private only	92.4	52.7	55.1	65.7
Other (none, Medicare, unknown)	3.3	22.5	16.0	16.4
AJCC stage				
*In situ*	17.0	19.3	22.1	18.7
Stage I	41.6	38.9	36.9	39.6
Stage II	29.7	28.3	26.7	28.6
Stage III	5.8	6.6	6.7	6.3
Stage IV	3.2	3.4	4.6	3.4
Unknown	2.7	3.6	3.1	3.3
Tumor size				
< 1 cm	20.0	20.0	23.4	20.2
1–< 2 cm	34.9	32.4	31.2	33.1
2–< 3 cm	18.5	16.9	15.0	17.3
3–< 4 cm	7.8	7.3	7.5	7.4
4+ cm	8.7	10.7	12.6	10.2
Other	3.2	3.3	2.9	3.2
Unknown	6.8	9.5	7.4	8.5
Lymph node involvement				
No nodal involvement	71.1	71.4	71.4	71.3
Positive nodes	25.5	24.9	24.3	25.1
Unknown	3.4	3.7	4.3	3.6
Histology				
Ductal	73.2	76.0	72.0	74.8
Lobular	17.2	14.3	16.3	15.4
Other	9.6	9.7	11.7	9.8
Neighborhood SES^b^				
Quintile 1 (lowest)	4.2	6.5	3.5	5.5
Quintile 2	11.3	11.1	7.2	10.8
Quintile 3	19.2	18.1	13.7	18.1
Quintile 4	29.1	25.4	22.5	26.4
Quintile 5 (highest)	36.2	39.0	53.2	39.1
% below poverty^c^				
0–4.9 %	45.9	45.5	45.8	45.7
5.0–9.9 %	26.8	25.3	27.5	26.0
10.0–19.9 %	19.2	19.0	17.4	18.9
≥ 20 %	8.1	10.2	9.5	9.5
Urban/rural				
Rural	4.6	6.9	4.9	6.0
Small towns	1.6	3.2	1.2	2.6
Small and medium size cities	29.2	29.4	7.6	27.8
Suburban metropolitan areas	53.5	48.9	57.7	51.1
Urban metropolitan areas	11.1	11.5	28.6	12.5
Population density^b^				
Quartile 1 (low density)	23.7	26.9	22.4	25.6
Quartile 2	31.2	31.1	25.5	30.8
Quartile 3	26.8	24.2	19.0	24.7
Quartile 4 (high density)	18.3	17.8	33.1	18.9
Hispanic ethnic enclave^b^				
Quintile 1 (low enclave)	22.7	25.1	24.8	24.3
Quintile 2	29.5	29.4	30.5	29.4
Quintile 3	27.6	24.7	25.7	25.7
Quintile 4	15.4	14.8	14.6	14.9
Quintile 5 (high enclave)	4.8	5.7	4.2	5.3
Unknown	0.3	0.3	0.1	0.3
Asian ethnic enclave^b^				
Quintile 1 (low enclave)	7.1	9.3	5.3	8.3
Quintile 2	16.2	16.9	13.3	16.5
Quintile 3	21.6	21.7	19.4	21.5
Quintile 4	24.9	24.0	23.5	24.3
Quintile 5 (high enclave)	29.8	27.8	38.4	29.2
Unknown	0.3	0.3	0.1	0.3
% Hispanic population^b^				
< 9 %	38.5	43.1	57.6	42.7
9–20 %	36.5	31.1	27.4	32.6
21–47 %	20.1	19.2	11.9	19.0
> 47 %	4.9	6.5	3.1	5.8
% non-Hispanic Asian population^b^				
< 2 %	9.1	11.4	6.4	10.3
2–4 %	22.4	22.4	16.1	22.0
5–12 %	29.0	29.0	28.2	29.0
> 12 %	39.5	37.2	49.3	38.7
% non-Hispanic White population^b^				
< 23 %	12.1	10.9	10.4	11.2
23–53 %	26.6	24.2	24.7	25.0
54–75 %	31.7	31.4	30.3	31.4
> 75 %	29.6	33.5	34.5	32.3
% non-Hispanic Black population^b^				
0 %	20.7	25.8	25.7	24.2
0.1–1.8 %	23.0	24.9	26.6	24.4
1.9–6 %	28.3	27.2	26.4	27.5
> 6 %	28.0	22.1	21.4	23.9

All comparisons are statistically different at *p* < .001 using Chi-squared tests with Bonferroni adjustment for multiple comparisons

*KPNC* Kaiser Permanente Northern California

^a^All frequencies (except for age) are age-adjusted to the age distribution of all cases. Includes counties of Alameda, Amador, Contra Costa, El Dorado, Fresno, Madera, Marin, Napa, Placer, Sacramento, San Francisco, San Joaquin, San Mateo, Santa Clara, Solano, Sonoma, and Yolo

^b^Quintiles or quartiles based on distribution of block groups in California; socioeconomic status based on composite of seven Census 2000 indicators for education, occupation, unemployment, household income, poverty, rent, and house values (Yost et al. [11]); Hispanic ethnic enclave based on Census data on linguistic isolation, English fluency, Spanish language use, Hispanic ethnicity, immigration history, and nativity; Asian ethnic enclave based on Census data on Asian/Pacific Islander race/ethnicity, language, nativity, and recency of immigration [16, 17, 19]

^c^Based on cut-off values from Krieger et al. [20, 24]

Compared with patients from all other hospitals, KPNC patients were less likely to reside in neighborhoods in the lowest and highest SES quintiles and more likely to represent middle SES neighborhoods (59.6 % versus 54.6 %), were more likely to live in neighborhoods characterized as suburban metropolitan areas (53.5 % versus 48.9 %), and in neighborhoods in the top two quartiles for population density (45.1 % versus 42.0 %). Proportionally more KPNC patients than patients from all other hospitals (all races/ethnicities combined) live in neighborhoods in the middle three Hispanic enclave quintiles (72.5 % versus 68.9 %); but slightly more KPNC patients live in Asian enclaves (54.7 % versus 51.8 % in top two quintiles for Asian enclaves). Accordingly, KPNC patients were more likely than patients from all other hospitals to live in neighborhoods with proportionally higher representation of non-White populations. These patterns also applied when comparing KPNC to all three groups combined (*N* = 112,244).

The 7 % of breast cancer patients reported from cancer centers differed substantially in patient demographic, clinical, and neighborhood characteristics compared with patients from the other two groups. Cancer center patients were proportionally more likely to be Asians/Pacific Islanders (16.0 % versus 13.0 % (KPNC) and 12.6 % (all other hospitals)), younger (31.1 % under age 50 versus 20.8 % (KPNC) and 23.9 % (all other hospitals)), and have more *in situ* (22.1 % versus 17.0 % (KPNC) and 19.3 % (all other hospitals)) and stages III and IV tumors (11.3 % versus 9.0 % (KPNC) and 10.0 %)). Cancer center patients also differed with regard to neighborhood factors. They were more likely to reside in the highest SES quintile (53.2 % versus 36.2 % (KPNC) and 39.0 % (all other hospitals)), suburban and urban metropolitan areas (86.3 % versus 64.6 (KPNC) and 60.4 % (all other hospitals)), and highest population density quartile (33.1 % versus 18.3 % (KPNC) and 17.8 % (all other hospitals)). Cancer center patients were comparable to patients from the other two groups for residence in Hispanic enclave but they were more likely to reside in high Asian enclave and high percentage Asian neighborhoods (49.3 % versus 39.5 % (KPNC) and 37.2 % (all other hospitals) for neighborhoods with >12 % Asian), and less likely to reside high Hispanic (15.0 % versus 25.0 % (KPNC) and 25.7 % (all other hospitals) for neighborhoods with >20 % Hispanics) and Black (21.4 % versus 28.0 % (KPNC) and 22.1 % (all other hospitals) for neighborhoods with >6 % Blacks) neighborhoods.

All comparisons were statistically different at *p* < .001 using Chi-squared tests with Bonferroni adjustment for multiple comparisons. A sensitivity analysis that included the 6 % (or 7567) of patients without census tract information resulted in similar results for the individual-level variables.

## Discussion

Using population-based cancer incidence data, we compared breast cancer patients diagnosed within KPNC, a large integrated health care system, which accounts for one-third of the breast cancer patient population in Northern California, to those from cancer centers (7 % coverage), and non-KPNC non-cancer center hospitals (61 % coverage). As expected, KPNC patients, by definition of their affiliation, were much more likely to have private health insurance than patients from other institutions. In comparison to non-KPNC, non-cancer center hospitals, we found that patients from KPNC differed somewhat by race/ethnicity (relatively fewer non-Hispanic Whites, but more non-Hispanic Blacks), stage at diagnosis (fewer *in situ*, but more stage I), neighborhood SES (proportionally fewer in lowest and highest SES quintiles), metropolitan areas (more likely to reside in suburban and urban metropolitan areas), population density (higher population density), and neighborhood racial/ethnic composition (slightly higher proportions of non-White residents). However, comparisons were statistically significant given the large sample sizes; differences were in fact modest, and sociodemographic and clinical characteristics were similar comparing the KPNC breast cancer patient population to other non-cancer center hospitals, despite the insurance differences.

To our knowledge, no prior research has assessed the representativeness of cancer patients from an integrated health care system to those from the underlying patient population, despite increasing interest in the use of EMR in research. One prior study, from 1985, of KPNC health plan members used SES measures from the 1980 Census [20] and showed that KPNC members were comparable to the underlying population with regards to racial/ethnic composition and percent working class, but were less likely to reside in lower SES neighborhoods as measured by percent below poverty and percent of adults with less than high school education. Because the earlier study considered binary cut-points for the three measures of neighborhood SES, it was not possible to determine whether fewer KPNC members resided in the highest SES neighborhoods.

In recent years, several internal KPNC reports have compared sociodemographic and selected behavioral risk factor information from the Kaiser Permanente Member Health Survey to 2007 and 2009 California Health Interview Surveys (CHIS) [21–23]. These reports show that KPNC members are of higher SES, include relatively fewer Hispanics and more non-Hispanic Whites, and have lower smoking prevalence among males than all non-members (including uninsured and those with public insurance). While KPNC members have similar behavioral and health risk factors, they were of slightly higher SES in terms of income and educational attainment (primarily among women) compared with non-members with private or government insurance. In comparison to all non-KPNC members regardless of insurance status, or to non-KPNC members with private or public insurance, KPNC members were representative of the highest SES groups when using individual- or household-level measures of educational attainment and income.

These findings differ from our results among female breast cancer patients showing KPNC patients were underrepresented in the highest SES quintile when using a composite, block group-level measure of SES. Our results may differ because the representativeness of KPNC breast cancer patients may be different than the representativeness of the general KPNC member population, representativeness may differ depending on the use of individual- versus neighborhood-level SES measures, and/or that our SES measure based on multiple SES indicators may provide more granularity in SES levels and thus enable a more accurate comparison. Regardless, in a cancer patient population, we found that KPNC breast cancer patients differed only modestly from patients in the underlying patient population with respect to sociodemographic, neighborhood, and clinical factors, and while some caution should be taken when generalizing results based on KPNC data to the underlying population of breast cancer cases, the KPNC population of breast cancer patients is generally representative of the Northern California population of breast cancer patients.

While breast cancer patients from NCI-designated cancer centers are a relatively small segment of the underlying patient population (7 %), they represent a significant proportion of clinical research findings reported in the literature. Yet, patients from the cancer centers were considerably different from patients from all other facilities in sociodemographic and clinical characteristics. Of note, the cancer center patients were from considerably higher SES neighborhoods than the other two groups of patients. To the extent that populations from integrated health care systems tend to be larger, coupled with the availability of EMR data, data from facilities like KPNC can provide the ability to generate data of relevance to minority and lower SES populations and provide insights into factors underlying health disparities.

It should be noted that comparisons for other cancers and/or health outcomes might be different than those based on breast cancer patients. However, comparable descriptive analyses can be conducted for other cancers or for other integrated health systems that provide care in areas with high-quality population cancer registries and that have similar richness of clinical information from EMRs. As our intent was to provide an assessment of comparability between different breast cancer populations by reporting facility type, we did not conduct multivariable analysis. Despite the descriptive nature of these analyses, our results should be informative to researchers using data pertaining to breast cancer from KPNC and perhaps other similar integrated health care systems.

## Conclusions

Given the modest differences in breast cancer patient characteristics comparing KPNC and all other facilities, integrated health care systems are likely more representative of the underlying population than academic medical centers, providing support for the generalizability of cancer research from this context.
